# What Causes Cancer Gallbladder?: A Review

**DOI:** 10.1155/1999/54515

**Published:** 1999-07

**Authors:** P. Chaurasia, M. K. Thakur, H. S. Shukla

**Affiliations:** 1 Bio-chemistry and Molecular Biology Laboratory Centre of Advanced Study in Zoology Banaras Hindu University Varanasi 221 005 India; 2 Division of Surgical Oncology Institute of Medical Sciences Banaras Hindu University Varanasi 221 005 India

## Abstract

Gallbladder cancer is a common malignancy of the biliary tract. It is the fifth common malignancy of the gastrointestinal tract in United States [1] and third in Northern India [2]. Despite such high prevalence, there is scanty published literature about this disease in indexed journals. Therefore, this article is intended to provide a brief overview of gallbladder cancer risk factors, based mainly on published evidence from analytical epidemiology and recent research findings of biologists and practising oncologists. Furthermore, an attempt has been made to establish an association between different causative factors and the occurrence of the disease.


*HPB Surgery*, 1999, Vol. 11, pp. 217-224
Reprints available directly from the publisher
Photocopying permitted by license only

© 1999 OPA (Overseas Publishers Association) N.V.
Published by license under
the Harwood Academic Publishers imprint,
part of The Gordon and Breach Publishing Group.
Printed in Malaysia.

### What Causes Cancer Gallbladder?: A Review

P. CHAURASIA[a], M. K. THAKUR[a] and H. S. SHUKLA[b,*]

[a] *Bio-chemistry and Molecular Biology Laboratory, Centre of Advanced Study in Zoology, Banaras Hindu University, Varanasi 221 005, India;*
[b] *Division of Surgical Oncology, Institute of Medical Sciences, Banaras Hindu University, Varanasi 221 005, India*

*(Received 10 May 1998; In final form 23 October 1998)*

**Gallbladder cancer is a common malignancy of the biliary tract. It is the fifth common malignancy of the gastrointestinal tract in United States [1] and third in Northern India [2]. Despite such high prevalence, there is scanty published literature about this disease in indexed journals. Therefore, this article is intended to provide a brief overview of gallbladder cancer risk factors, based mainly on published evidence from analytical epidemiology and recent research findings of biologists and practising oncologists. Furthermore, an attempt has been made to establish an association between different causative factors and the occurrence of the disease.**

*Keywords:* Gallbladder cancer, risk factors

### PATHOLOGY

Gallbladder cancer is usually present as a localized extensive tumor. The whole gallbladder may appear thickened, lobulated, and contracted into the liver. Occasionally it may be filled with purulent material mucus or stones which may cause extensive hemorrhage and necrosis and sometimes even lead to perforation. Carcinoma is most often located in the fundus and may be present as a mucosal plaque, a polypoid or papillary excrescence or discrete thickening of wall, sometimes with an attached gallstone [3-5].

Gallbladder cancer has several histopathological features. In the majority of cases, it is an adenocarcinoma (85-90%) which can be papillary, nodular, tubular or a combination with varying degrees of invasions. The histopathological grade and degree of metaplasia have prognostic significance, whereas other histopathological types are undifferentiated or anaplastic carcinoma (2-7%), pure squamous cell carcinoma (1-6%) and adenosquamous carcinoma (1-4%) [6,7]. Rare types of gallbladder cancer include malignant melanomas, lymphomas, sarcomas and carcinoid tumors. Fahim [8] explained different modes of spread of gallbladder cancer including direct, lymphatic, vascular, neural, intraperitoneal and intraductal. Nodular tumors are more likely to infiltrate early, invade the liver and spread to adjacent lymph nodes than papillary tumors.

At an advanced stage, the gallbladder is unresectable in most patients. It is difficult to diagnose malignancy at an early stage due to its

*Corresponding author. Tel.: 0091 542 311978, Fax: 0091 542 313965, e-mail: hsshukla@banaras.ernet.in nonspecific symptoms. However, recent advances in preoperative imaging of early gallbladder malignancy and its radical aggressive surgery leads to improvement in survival [9].

### GEOGRAPHICAL DISTRIBUTION

Gallbladder cancer is a relatively rare neoplasia in the world and its incidence shows considerable geographical variation. The complete ascertainment of all previously diagnosed cases of gallbladder cancer in a defined area is a difficult task. However, such data have been collected from specific areas in different time period. In a population-based case-control study of cancer of bile ducts and gallbladder in Greater Montreal between 1984 and 1988, a total of 24 patients with cancer of bile ducts and 33 patients with cancer of gallbladder were compared to 239 population-based controls. Gallbladder cancer risk was found to be eight times more in those having previous gallbladder problem than controls [10].

The Surveillance, Epidemiology and End Results (SEER) program of National Cancer Institute (NCI) of United States provides data indicating racial and ethnic variations in the incidence of gallbladder cancer in United States. The average annual age-adjusted incidence rates between 1975–1985 per 100,000 for selected gallbladder cancer cases varied with sex between racial and ethnic groups. The incidence rate among males was 0.8 White, 0.8 Black, 1.5 Hispanics, 8.9 American Indians, 1.2 Chinese, 1.5 Japanese, 1.2 Filipinos and 1.4 Hawaiians, whereas among females it was 1.6 White, 1.1 Black, 7.1 Hispanics, 17.1 American Indians, 1.0 Chinese, 1.7 Japanese, 1.8 Filipinos and 1.3 Hawaiians. In United States, the incidence is highest in New Mexico accounting 8.3% of all cancers. In Israel, a very high rate of gallbladder cancer includes 13.8 per 100,000 females and 7.5 per 100,000 males. Similar to United States, wide variations in incidence rates exist throughout the world [11]. The incidence rate also varies within the Indian population. It is very high in Northern India, 4.5 per 100,000 for males and 10.1 per 100,000 for females, but low in South India, 1.2 per 100,000 for males and 0.9 per 100,000 for females as reported by the population-based cancer registry, Delhi [12]. Other countries with high rates of gallbladder cancer include Bolivia, Chile and Northern Japan. Such variation in incidence rate is influenced by genetic, environmental, diet and socioeconomic factors [11].

Moreover, migration from native land also affects the incidence rate of gallbladder cancer. Swerdlow [13] compared the risk of cancer mortality (during 1973 to 1985) in persons who were born in Indian subcontinent but migrated to England and Wales, with the ethnic population of the country, for various kinds of cancer. There was a substantial increased risk of gallbladder cancer in females of Indian ethnicity than other British ethnic migrants.

Descriptive epidemiology is used to investigate the occurrence of gallbladder cancer in different population groups. The incidence rate of gallbladder cancer can be derived only from population-based cancer registries. However, the number of such registries in the world has increased steadily over the last 50 years, but in comparison with the availability of data on mortality, the population so covered remains limited. Furthermore, the populations served by cancer registries often comprise only the inhabitants of a major city and do not represent the country as a whole.

### AGE

In general, the incidence of gallbladder cancer rises with advancing age. The enhanced susceptibility to cancer may result from the impairment of gene and immune functions associated with later stages of the life span.

Paraskevopoulos [14] reported that gallbladder cancer incidence rate increases progressively with age, particularly after the fifth decade. Sipetic [15] analysed the epidemiological situation of chosen malignant tumors of digestive tract for the period 1960–1990 in Serbia. Based on mortality rate, it was reported that more women died of gallbladder cancer than men (1.8 : 1). In both male and female population, the highest average specific mortality rate was observed for a person over the age of 75 years.

### SEX

As evident from medical reports, the prevalence of gallbladder cancer is higher in females than males. According to SEER program of NCI of United States, the incidence of gallbladder per 100,000 is higher among white females than males between 1981–1985. The incidence and mortality rates for primary gallbladder cancer in white males and females were 1.6 and 1.1, respectively [11]. About 75% of female patients are in their seventies and their diagnosis is made during surgical exploration. In more than three-fourth patients, gallbladder cancer is associated with cholelithiasis [1, 16–18].

A retrospective study of 14,018 malignant tumors from Northern Pakistan reflects differential pattern of cancer distribution among different ethnic groups like Pathans, Punjabis and others. When the standardized cancer ratio (ASCAR) was calculated to compare the data from the neighbouring countries of the region, higher frequency was found for the prostate cancer in males and gallbladder carcinoma in females [19].

Rougereau and co-workers [20] recorded 77 cases of primary gallbladder carcinoma in Calvados Digestive Tumors Registry covering a population of 589,559 inhabitants between 1978–1986. The crude incidence was 2.4/100,000 in females and 0.6/100,000 in males (sex ratio 3.8). Similarly, 56 patients with carcinoma of gallbladder were treated during the year 1969–1987 at the Beth Israel Medical Centre, New York, USA. The disease was found to be most common among elderly females with cholelithiasis [21].

### SEX HORMONES

The preponderance of gallstones in women [20] suggests that female sex hormones play a role in the pathogenesis of gallstones which are present in a majority of cases of gallbladder cancer [21]. Chen and Huminer [22] found high level of estrogen and its receptor (ER) in gallbladder cancer. Nakamura and co-workers [23] carried out immuno-histochemical examination of cancerous tissues from 21 patients with primary gallbladder cancer and observed nuclear localization of ER in 52.4% patients. However, moderately and poorly differentiated adenocarcinoma have a relatively higher tendency of ER-positivity than well differentiated adenocarcinoma. The ER associated protein p29 and estrogen induced protein pS2 were expressed in cancer gallbladder tissues [23]. Early tumors had a significantly lower expression of p29 than advanced tumors and metastases [24]. Furthermore, the relationship between combined oral contraceptive and primary gallbladder cancer was examined in 58 cases and 355 controls participating in an international hospital based case-control study. Use of combined oral contraceptive at any time was not associated with the risk of developing gallbladder cancer. There was also no evidence that the oral contraceptives caused risk of gallbladder cancer in women with or without *a prior* history of gallbladder disease. A history of gallbladder disease or gallstones was a strong risk factor for gallbladder cancer [25]. Epidemiological studies also show a strong association between gallbladder cancer, obesity and estrogen [26, 27].

Recently Lichtman [25] examined pre-menopausal and post-menopausal females into their fifth decade and found gallbladder disease and breast cancer as the possible side effects of hormone replacement therapy.

### GENETIC FACTORS

Multistep models of carcinogenesis require a series of genetic and epigenetic events to occur during both the initiation and progression of malignancy. While the precise nature of all steps leading to malignancy remains unknown, various oncogenes and tumor suppressor genes are sequentially altered either through mutations or deletions [28,29]. Kamel and co-workers [30] found that mutations in p53 and c-erbB-2 play a major role in neoplastic transformation of gallbladder epithelial cells. Co-expression of p53 and c-erbB-2 suggests that alterations of these genes might act in concert during the neoplastic transformations. The expression of p53 in gallbladder dysplasia suggests that p53 mutation could be an early event in the evolution of some gallbladder carcinomas. The study of Wee *et al.* [31] also supported the notion that p53 mutation may have a role in the pathogenesis of gallbladder cancer. Similarly, Yabar and Yatanabe [32] reported overexpression of p53 gene and protein in adenocarcinoma of gallbladder with a typical epithelium. Current histopathological evidence suggests that two distinct pathways are involved in gallbladder carcinogenesis: (1) *de novo* carcinoma develops from a predominant p53 alteration with low K-ras mutation, and (2) carcinoma in pyloric gland type adenoma develops from alteration in p53, K-ras and APC genes [33].

Wistuba *et al.* [34] investigated the incidence of ras gene mutations and loss of heterozygosity (LOH) at p53, DCC and Rb genes and 5q, 3p, 8p and 9p loci from 25 gallbladder carcinomas in Chile. LOH of p53 occurred more frequently and earlier than protein overexpression. Todoroki and co-workers [35] detected point mutations in p16InK4/CDKN2 gene (a regulatory gene of mammalian cell cycle) resulting in primary carcinoma of biliary tract. This includes 15 missense and 2 silent mutations. The frequency of mutations in gallbladder cancer and hilar bile duct cancer were 80% (8 of 10) and 63% (5 of 8), respectively. Recently, a src-related signal pathway represented the role of activated ras and src oncogene products in the acquisition of fully neoplastic phenotype by human gallbladder adenocarcinoma cells [36]. The expression of c-fos protein plays an important role in the development of gallbladder neoplasia with increased immunoreactivity in gallbladder cancer but not in chronic cholecystitis, biliary tract and ampullary neoplasms [37]. Oncogenic K-ras codon 12 mutation was detected in 59% (30 of 51) of gallbladder carcinomas, 73% (8 of 11) of gallbladder dysplasia and in gallstones. This is an important precancerous lesion of gallbladder carcinogenesis [38]. Sasatomi *et al.* [39] found an increased frequency of apoptosis with the progression of gallbladder carcinoma. Oncogenic alterations of bcl-2 and p53 may also play a role in tumorigenesis of gallbladder carcinoma, but these changes had no correlation with spontaneous apoptosis [39].

### CHOLELITHIASIS

A history of gallbladder disease or gallstones is a strong risk factor for gallbladder cancer. In ultrasound findings, 4 out of 5 cases of primary carcinoma of the gallbladder had associated gallstones [40]. Gallstones or a previous cholecystectomy for gallstones was reported in 44% of women and 23% of men in a postmortem examination of patients in the Canton of Thrgau between 1989 – 1991. Among the gallstones carriers, 8% of women and 2.5% of men had developed a gallbladder carcinoma [41]. An interracial study has been carried out with respect to gallstone size, growth and the relation between stone size and gallbladder cancer. The prevalence of gallstones differs markedly among 1676 females (169 White, 531 Black and 976 American Indians). One-third of all gallbladder cancers in subjects with calculi was associated with large stones [42]. Thus the stone size might be used to determine the risk of gallbladder cancer in patients with gallstones. Gallbladder cancer is detected in about one percent of patients undergoing cholecystectomy for gallstones in some studies [1, 43, 44].

Bllesta-Vicente and co-workers [45] reviewed retrospectively 120 patients with histologically diagnosed gallbladder carcinoma during a period of 16 years (1974–1989). The results showed that (i) the annual incidence of gallbladder carcinoma remains unchanged; (ii) there is a close association of cancer with gallstone; (iii) the late diagnosis is due to non-specificity of clinical manifestations and (iv) early gallbladder carcinoma is only diagnosed by chance in cholecystectomy specimens. Calvados Digestive Tumour Registry recorded 77 primary gallbladder carcinomas out of 125 extra-hepatic bile duct carcinomas between 1978 and 1986 [20]. However, out of 74 operated patients gallbladder carcinoma was diagnosed preoperatively in only 13.5%, cholelithiasis was found to be associated with 81.3%.

Nadler and McSherry [21] reviewed 56 patients with carcinoma of gallbladder treated during the 17-year period 1969–1987 at the Beth Israel Medical Centre, New York, and found cholelithiasis as the major causative agent of gallbladder carcinoma among elderly women.

### CHOLECYSTECTOMY

The risk of malignancy associated with calcified or porcelain gallbladder is about 20% [46]. Zhou [47] demonstrated post operative recurrence of gallbladder carcinoma. The study was divided into two groups namely A and B. Group A (48 cases) represents patients dying within one year of operation and Group B (23 cases) represents those who remain alive for more than three years. Clinico-pathological, morphological and histological examinations showed statistically significant differences between the two groups. Risk factors relating to early post-operative recurrence of gallbladder carcinoma included many factors like, deeply involved liver parenchyma (> 75 mm in depth), cervical cancer and palliative resection.

### ABO BLOOD GROUPS

A high incidence of various cancers like gastric, salivary, colon, pancreas, kidney, bladder, uterus, ovary and cervix in blood group A has been well documented.

Juvonen and Niemela [48] analysed ABO blood group in 171 patients with symptomatic gallstones. However, they could not draw any significant correlation between formation of gallstones and different blood groups except blood group A who had multiple stones.

Similarly in a mirror image paper, Pandey *et al.* [49] studied the distribution of ABO and Rh blood groups in 69 patients with gallbladder cancer and 152 patients with cholelithiasis registered at the Banaras Hindu University hospital. Like other carcinomas, an increased frequency of gallbladder carcinoma was found in blood groups A and AB when compared with the control population. This appears to be due to higher formation of gallstones in blood group A population. An increased risk of development of gallbladder cancer in patients with blood group A has been attributed to the expression of Forssmann antigen in these patients. Forssmann antigen has structural similarity with blood group antigen A [49].

Thus antibodies to blood group A probably attach precancerous and cancerous cells expressing Forssmann antigen. Blood group A and AB lack antibodies to A and hence are theoretically more susceptible to develop these carcinomas.

### CARCINOGENS

As in other carcinomas, the role of carcinogens in the involvement of gallbladder cancer cannot be ruled out. It has been reported that methylcholanthrene, O-aminoazotoluene and nitrosamines cause gallbladder cancer in hamsters [50]. It is likely that adulteration of mustard oil with some carcinogen acts as an aetiological factor for high incidence of gallbladder cancer in North India [46]. Increased prevalence of gallbladder cancer has also been related to specific occupations. For example, Paraf *et al.* [51] reported the case of a 64-year old woman having squamous cell carcinoma of the gallbladder with hepatic metastases due to exposure to trichloroethylene in a degreasing metal laboratory, although trichloroethylene could not be assumed as a true carcinogen for gallbladder cancer. A high incidence of gallbladder cancer has been reported in individuals working in the rubber industry and animal studies suggest that azotoluene and nitrosamines are potent chemical carcinogens [1].

### MISCELLANEOUS FACTORS

### 1. Anomalous Pancreatic Biliary Duct Junction (APBDJ)

APBDJ allows reflux of pancreatic juice into gallbladder leading to precancerous changes in gallbladder mucosa [52]. Kimura *et al.* [53] reported APBDJ in 17% patients of gallbladder cancer but in only 3% of other hepatobiliary disorders. Patients with choledochal cysts are more prone to develop gallbladder carcinomas.

### 2. Typhoid Infection

Salmonella typhi infection has been found to be associated with an increased risk of gallbladder cancer and bile duct cancers [1, 54]. There exists no such correlation in Indian subcontinent where typhoid infection is a common occurrence.

### 3. Rare Lesions

Mirizzi syndrome is a rare complication of longstanding cholelithiasis. An elevated level of tumor associated antigen CA 19-9 are indicative of a coincidental gallbladder carcinoma [55]. Segmental adenomyomatosis of gallbladder, chronic inflammatory bowel disease [56] and polyposis coli [57] are rare associations with gallbladder cancer.

### CONCLUSION

Although abundant data concerning clinical manifestation of gallbladder cancer have accumulated, the genetic features of neoplasm still remain ill-defined. In addition to genetic factors, research based on several risk factors should be undertaken to resolve issues relating to geographical variation, incidence rate, food habit, optimal surgical and multidisciplinary management of gall bladder cancer.

However, the possibility for preventing mortality from this disease through early detection of genetic markers may be possible in the future. Recent advances in molecular biology will help in understanding the mechanisms responsible for development of biological heterogeneity in gallbladder cancer, the pathogenesis of invasion and metastasis, and ultimately improved management of the disease and perhaps its prevention.

### *References*

[1] Pitt, A., Dooley, W. C., Yeo, C. J. and Cameron, J. L. (1995). Malignancies of the biliary tree. *Curr. Probl. Surg.*, **32**,1.

[2] Shukla, V. K., Khandelwal, C., Roy, S. K. and Vaidya, M. P. (1985). Primary carcinoma of the gallbladder: A review of 16 year period at University Hospital. *J. Surg. Oncol.*, **28**, 32–35.

[3] Frierson, H. F. and Fechnes, R. E. (1987). Pathology of malignant neoplasms of the gallbladder and extrahepatic bile ducts: In: Wanebo, H. J. (Ed.): Hepatic and Biliary cancer, Marcel Dekker, New York, pp. 281–297.

[4] Edmonsdon, H. A. (1967). Tumors of the Gallbladder and Extrahepatic Bile Ducts, Section VII, Fascicle 26, Washington DC, Armed Forces Institute of Pathology.

[5] Tyagi, S. P., Tyagi, N., Maheshwari, V., Ashraf, S. M. and Sahoo, P. (1992). Marphological changes in diseased gallbladder: a study of 415 cholecystectomies at Aligarh. *J. Indian Med. Assoc.*, **90**, 178–181.

[6] Albores-Saavedra, J., Henson, D. E. and Sobin, L. H. (1992). The WHO histologica; classification of tumors of the gallbladder and extrahepatic bile duct. A commentary on the second edition. *Cancer*, **70**, 410–414.

[7] Henson, D. E., Albores-Saavedra, J. and Corle, D. (1992). Carcinoma of the gallbladder. Histologic types, stage of disease, grade and survival rates. *Cancer*, **70**, 1493–1497,

[8] Fahim, R. B., McDonald, J. R., Richards, J. C. *et al.* (1962). Carcinoma of the gallbladder: A study of its modes of spread. *Ann. Sur.*, **156**, 114–124.

[9] Shukla, H. S., Avasthi, K. and Naithani, Y. P. (1981). A clinicopathological study of carcinoma gallbladder. *Ind. J. Cancer*, **18**, 198–200.

[10] Ghadirian, P., Simard, A. and Baillargeon, J. (1993). A population-based case-control study of cancer of bile ducts and gallbladder in Quebec, Canada. *Revue De Epidemiologie Et De Sante Publique*, **42**, 107–112.
[11] Fraumeni, J. F. Jr., Hoover, R. N., Devesa, S. S. and Kinlen, L. J. (1989). Epidemiology of cancer, In: DeVita V. T. Jr. (Ed.) *Cancer: Principles and Practice of Oncology*; Philadelphia, J. B. Lippincott Company, pp. 198–227.
[12] Indian Council of Medical Research (ICMR) Annual Report of population-based cancer registries of the National cancer Registry Programme (1996) New Delhi, ICMR publication, p. 18.
[13] Swerdlow, A. J., Marmot, M. G., Grulich, A. E. and Head, J. (1995). Cancer mortality in Indian and British ethnic immigrants from the Indian subcontinent to England and Wales. *Br. J. Cancer*, **72**, 1312–1319.
[14] Paraskevopoulos, J. A., Dennisor, A. R. and Johnson, A. G. (1991). Primary carcinoma of gallbladder. *HPB Surgery*, **4**, 277–289.
[15] Sipectic, S., Jarebinski, M., Vlajinac, H., Adanja, B. and Vojnosanit-Pregl, A. (1995). Epidemiologic characteristics of malignant tumors of the digestive tract in Serbia 1969–1990. *Serbo-Croatian-Roman*, **52**, 359–364.
[16] Nevin, J. E., Moren, T. J., Kay, S. *et al.* (1976). Carcinoma of the gallbladder. *Cancer*, **37**, 141–148.
[17] Wanebo, H. J., Castle, W. N. and Fechner, R. E. (1982). Is carcinoma of the gallbladder a curable lesion? *Ann. Sur.*, **195**, 624–631.
[18] Morrow, C. E., Sutherland, D. E., Fiorack, G. *et al.* (1983). Primary gallbladder: Significance of suberosal leisons and results of aggressive surgical treatment and adjuvant chemotherapy. *Surgery*, **94**, 709–714.
[19] Ahmed, M., Khan, A. H. and Mansoor, A. (1991). The pattern of malignant tumours in northern Pakistan, *J. Pak. Med. Assoc.*, **41**, 270–273.
[20] Rougereau, A., Verwaede, J. C. and Gignoux, M. (1993). Cancer of gallbladder, Epidemiology, Diagnosis and prognostic factor. *Annales De Chirugie*, **47**, 18–23.
[21] Nadler, L. H. and McSherry, C. K. (1992). Carcinoma of the gallbladder: review of the literature and report on 56 cases at the Beth Isreal Medical Centre. *Mount Sinai J. Med.*, **59**, 47–52.
[22] Chen, A. and Huminer, D. (1991). The role of estrogen receptors in the development of gallstones and gallbladder cancer. *Med. Hypo.*, **36**, 259–60.
[23] Nakamura, S., Muro, H. and Suzuki, S. (1989). Estrogen and progesterone receptors in gallbladder cancer. *Jap. J. Cancer.*, **19**, 189–194.
[24] Roa, I., Araya, J. C., Villaseca, M., de-Artxaballa, X., Ferreira, A. and Roa, J. C. (1995). Gallbladder cancer: Immunohistochemical expression of the protein related to estrogen receptor (p29) and of the protein induced by estrogen (pS2). *Rev. Med. Chil.*, **123**, 1333–1340.
[25] Lichtman, R. (1996). Premenopausal and postmenopausal hormone replacement therapy Part I An update of literature on benefits and risks. *J. Nurse Midwifery*, **41**, 3–28.
[26] Hasegawa, R., Ogawa, K., Takaba, K., Shirai, T. and Ito, N. (1992). 3,2′-Dimethyl-4-aminobiphenyl induced gallbladder carcinogenesis and effects of ethinyl estradiol in hamsters. *Jpn. J. Cancer Res.*, **83**, 1286–1292.
[27] Combined oral contraceptives and gallbladder cancer. The WHO collaborative study of Neoplasia and steroid contraceptives. *1989 Int. J. Epidemiol.*, **18**, 309–314.
[28] Fearon, E. R. and Vogelstein, B. (1990). A genetic model for colorectal tumorigenesis. *Cell*, **61**, 759–767.
[29] Vogelstein, B. and Kinzler, K. W. (1993). The multiple nature of cancer. *Trends Genet*, **9**, 138–141.
[30] Kamel, D., Paakko, P., Nuorva, K., Vahakangas, K. and Soini, Y. (1993). p53 and c-erb-2 protein expression in adenocarcinomas and epithelial dysplasias of the gallbladder. *J. Pathol.*, **170**, 67–72.
[31] Wee, A., Teh, M. and Raju, G. C. (1994). Cliical importance of p53 proteins in gallbladder carcinoma and its precursor lesions. *J. Clin. Pathol.*, **47**, 453–56.
[32] Yabar, H. A. and Wayanabe, H. (1994). An early carcinoma of the gallbladder with atypical epithelium (a report of case with elevated p53 expression). *Rev Gastroenterol Peru*, **14**, 65–68.
[33] Itoi, T., Watanabe, H., Ajioka, Y., Oohashi, Y., Takel, K., Nishiura, K., Nakamura, Y., Horil, A. and Saito, T. (1996). APC, K-ras codon 12 mutations and p53 gene expression in carcinoma and adenoma of the gallbladder carcinogenesis. *Pathol. Int.*, **46**, 333–340.
[34] Wistuba, I. I., Sugio, K., Hug, J., Kishioto, Y., Virmani, A. K., Rao, I., Albores-Saavedra, J. and Gazdar, A. F. (1995). Allele-specific mutations involved in the pathogenesis of endemic gallbladder carcinoma in Chile. *Cancer Res.*, **15**, 2511–2515.
[35] Todoroki, T., Ichikawa, Y., Hanai, S., Suzuki, H., Hori, M., Fukao, K., Miwa, M. and Uchida, K. (1995). Mutations of p16Ink4/CDKN2 and p15Ink4B/MTS2 genes in biliary tract cancers. *Cancer Res.*, **55**, 2756–2760.
[36] Tatusumoto, T., Nakano, S., Shimizu, K., Ono, M., Esaki, T., Ohshima, K. and Niho, Y. (1995). Direct tumorigenic conversion of human gallbladder carcinoma cells by v-src but not by activated c-H-ras oncogene. *Int. J. Cancer*, **61**, 206–213.
[37] Lee, C. S. (1996). Differences in c-fos gene product expression in cancers of the gallbladder and biliary tract. *Pathol. Int.*, **46**, 771–776.
[38] Ajiki, T., Fujimori, T., Onoyama, H., Yamamoto, M., Kitazawa, S., Maeda, S. and Saitoh, Y. (1996). K-ras gene mutation in gallbladder carcinomas and dysplasia. *Gut.*, **38**, 426–429.
[39] Sasatomi, E., Tokunaga, O. and Miyazaki, K. (1996). Spontaneous apoptosis in gallbladder carcinoma. Relationships with clinicopathologic factors, expression of E-cadherin, bcl-2 ptotooncogene, and p53 oncosuppressor gene. *Cancer*, **78**, 2101–2110.
[40] O'Keeffe, F., Lorigan, G. and Butler, F. (1989). Ultrasound findings in carcinoma of the gallbladder. *Iri. J. Med. Sci.*, **158**, 48–49.
[41] Etspuler, H. K., Asper, R., Wild, J. A. and Hardmeier, T. (1993). Analysis and epidemiology of gallstones in decreased and autopsied patients of the Thuragu Canton. *Ther-Umsch.*, **50**, 535–540.
[42] Lowenfels, A. B., Walker, A. M., Althaus, D. P. *et al.* (1989). Gallstones growth, size and risk of gallbladder cancer: an interracial study. *Int. J. Epidemiol.*, **18**, 50–54.
[43] Hamrick, R. E. Jr., Liner, F. J., Hastings, P. R. *et al.* (1982). Primary carcinoma of gallbladder. *Ann Sur.*, **195**, 270–273.
[44] Wanebo, J. H. and Vezeridis, M. P. (1993). Carcinoma of the gallbladder. *J. Sur. Oncol.*, (Suppl. 3), pp. 134–137.
[45] Ballesta-Vicente, Castellano, G. and Colina, F. (1991). Sixteen-year experience with gallbladder. Review of 120 cases. *Revista Epanola de Enfermedades Digestivas*, **79**, 324–330.
[46] Misra, N. C., Misra, S. and Chaturvedi, A. (1997). Carcinoma of gallbladder: In: Taylor, I. and Johnson C. D. (Ed.): *Recent Advances in Surgery*, Churchill Livingstone. pp. 69–88.

[47] Zhou, Z. (1993). The clinicopathological research of post-operative recurrence of gallbladder carcinoma. *Chung Hua Wai Ko Tsa Chih.*, **31**, 477–479.

[48] Juvonen, T. and Niemela, O. (1992). ABO blood groups and gallstone diseases. *Br. Med. J.*, **305**, 26–27.

[49] Pandey, M., Gautam, A. and Shukla, V. K. (1995). ABO and Rh groups in patients with cholelethiasis and carcinoma of the gallbladder. *Br. Med. J.*, **310**, 1639.

[50] Kowalewski, K. and Todd, E. F. (1971). Carcinoma of the gallbladder induced in hamsters by insertion of cholesterol pellets and feeding dimethylnitrosamine. *Proc. Soc. Exp. Biol. Med.*, **136**, 482–86.

[51] Paraf, F., Paraf, A. and Barge, J. (1990). Are industrial toxic substances risk factor in gallbladder cancer? *Gastroenterologie Clinique Et Biologique*, **14**, 877–880.

[52] Mori, K., Nagakawa, T., Ohta, T. *et al.* (1993). Association between gallbladder cancer and anomalous union of the pancreatiobiliary ductal system. *Hepatogastroenterology*, **40**, 56–60.

[53] Kimura, K., Ohta, M., Saisho, H. *et al.* (1985). Association of gallbladder carcinoma and anamolous pancreaticobiliary ductal union. *Gastroenterology*, **89**, 1258–1265.

[54] Redaelli, C. A., Buchler, M. W., Schilling, M. K., Krahenbuhl, L., Ruchti, C., Blumgart, L. H. and Baer, H. U. (1997). High coincidence of Mirizzi syndrom and gallbladder carcinoma. *Surgery*, **121**, 58–63.

[55] Axelord, I., Munster, A. M. and O'Brien, T. H. (1979). Typhoid cholecystisis and gallbladder cancer after an interval of 67 years. JAMA, **217**, 83.

[56] O', Connor, Harding, B., Green, D. and Coolian, J. (1986). Primary carcinoma of the gallbladder associated with ulcerative colitis. *Postgard Med. J.*, **62**, 871–872.

[57] Willson, S. A., Princenthal, R. A., Law, B. and Leopold, G. R. (1987). Gallbladder carcinoma in association with polyposis coli. *Br. J. Radiol.*, **60**, 771–773.

